# Extended Injection Intervals after Switching from Ranibizumab to Aflibercept in Macular Edema due to Central Retinal Vein Occlusion

**DOI:** 10.1155/2018/8656495

**Published:** 2018-07-15

**Authors:** Sylvia Nghiem-Buffet, Agnès Glacet-Bernard, Manar Addou-Regnard, Eric H. Souied, Salomon Y. Cohen, Audrey Giocanti-Auregan

**Affiliations:** ^1^Centre Ophtalmologique d'Imagerie et de Laser, 11 Rue Antoine Bourdelle, Paris, France; ^2^Ophthalmology Department, DHU Vision and Handicaps, APHP, Avicenne Hospital, Paris 13 University, 125 Rue de Stalingrad, Bobigny, France; ^3^Department of Ophthalmology, Centre Hospitalier Intercommunal, Université Paris-Est-Créteil, Paris 12 University, 40 Avenue de Verdun, 91000 Créteil, France

## Abstract

**Purpose:**

To assess treatment interval extension after switching from ranibizumab to aflibercept intravitreal injections in macular edema (ME) due to central retinal vein occlusion (CRVO) with an insufficient response or frequent recurrences to initial treatment.

**Methods:**

CRVO eyes treated with ranibizumab injections on a treat-and-extend (TAE) basis with an insufficient response or frequent recurrences were switched to aflibercept. Primary endpoint was the change in injection intervals before and after the switch.

**Results:**

Eleven eyes were included in this retrospective bicentric study. Before switching, patients received a mean number of 15.3 ranibizumab injections (range, 6–34) during a mean follow-up of 23.4 months (range, 6–57). After switching to aflibercept, patients received a mean number of 12.4 injections (range, 6–20) during a mean follow-up of 25.5 months (range, 16–38). Treatment interval could be extended from 6.1 (range, 4–8) to 11 weeks (range, 8–16) (*p*=0.001) corresponding to a mean extension of injection interval of +4.9 weeks.

**Conclusion:**

In case of insufficient response or frequent recurrences of ME due to CRVO in patients treated with ranibizumab on a TAE basis, switching to aflibercept could allow extending treatment intervals, which could reduce the injection burden for these patients.

## 1. Introduction

Macular edema (ME) is the leading cause of vision loss in case of central retinal vein occlusion (CRVO). The prognosis of this disease has recently been considerably improved through the use of intravitreal injections of anti-VEGF agents [1–4] or corticosteroids [5, 6].

ME secondary to CRVO is mainly due to an abnormal vascular permeability involving the vascular endothelial growth factor (VEGF) [7]. Anti-VEGF therapy is one of the treatments available for ME since 2011: in 2011, ranibizumab (Lucentis) has been approved by the Food and Drug Administration (FDA) following the CRUISE study [8], and then aflibercept (Eylea) has been approved for the treatment of ME due to CRVO in 2013 following the GALILEO [1] and COPERNICUS [3] phase 3 clinical trials. In France, both ranibizumab and aflibercept are covered by the healthcare system.

Thus, the first anti-VEGF treatment prescribed in our practice for ME secondary to CRVO was ranibizumab because of its earlier availability. The RETAIN study [9] has shown that among patients with ME due to CRVO well managed with ranibizumab, 56.2% of patients still required frequent retreatment for the third and the fourth year of follow-up. In these particular cases in which the response to ranibizumab seems insufficient or when an extension of treatment intervals is not possible, a switch to another drug may help to manage ME and to reduce treatment burden for patients. Clinicians may either change pharmaceutical class and use dexamethasone implant or switch to another anti-VEGF treatment. Some studies have reported results supporting the efficacy of switching to aflibercept after ranibizumab failure [10–14].

Unlike ranibizumab, aflibercept binds not only to VEGF-A but also to VEGF-B and placental growth factor (PlGF) [15]. This different mechanism of action may explain the possible efficacy of aflibercept after ranibizumab failure even if they belong to the same therapeutic class.

The aim of this study was to assess the extension of treatment intervals after switching from ranibizumab to aflibercept intravitreal injections in recurrent ME and in ME with insufficient response to initial treatment in CRVO.

## 2. Methods

A retrospective study was conducted in two tertiary centers specialized in imaging and treatment of retinal diseases (Centre Ophtalmologique d'Imagerie et de Laser in Paris and the Department of Ophthalmology of the Intercommunal Hospital Center in Créteil, France). All consecutive patients treated with ranibizumab on a TAE regimen for ME due to CRVO and switched to aflibercept between January 2014 and December 2015 were included. The TAE schedule consisted of an initial intravitreal ranibizumab loading dose of three consecutive monthly injections, followed by monthly injections until the macula was dry on SD-OCT. The interval between treatments was extended by fixed 2-week increments if no exudative changes were observed on SD-OCT. In case of fluid recurrence on SD-OCT, the interval was reduced back down by one or two weeks.

The decision to switch to aflibercept was at the discretion of one of the investigators (SNB and AGB) in case of frequent recurrences or insufficient response to ranibizumab defined in this study as follows:

  Frequent recurrences were defined by a maximum relapse-free interval of 8 weeks or less. The patients were considered to have frequent recurrences of ME due to CRVO, although it was controlled with ranibizumab, if the interval between injections was less than or equal to 8 weeks. The interval between injections was based on a TAE regimen. This interval was the maximum interval needed to prevent ME recurrence and to stabilize visual acuity.
  Insufficient response to ranibizumab treatment was defined by a reduction in central retinal thickness (CRT) but persistence of fluid or cystoid change in the central subfield despite at least 6 monthly ranibizumab injections with the impossibility to extend interval more than 4 weeks. Persistent ME was defined on SD-OCT (Cirrus 5000, ZEISS Meditec, Germany; Spectralis, Heidelberg, Germany) by a loss of the foveal pit and a CRT >300 *µ*m.

This study was conducted in accordance with the tenets of the Declaration of Helsinki, and an informed consent was obtained from all patients. Approval was obtained from the Federation France Macula ethics committee.

Inclusion criteria were as follows:

- Patients aged at least 18 years
- Patients with ME due to CRVO treated with ranibizumab on a TAE regimen with frequent recurrences or insufficient response to treatment
- Patients with a minimum follow-up of twelve months after the switch to aflibercept.

Exclusion criteria were as follows: other ocular conditions impairing vision, fewer than six ranibizumab injections prior to the switch to aflibercept, and incomplete imaging or clinical data.

All patients underwent a complete ophthalmological examination before switching including medical history and comorbidities (high blood pressure), best-corrected visual acuity (BCVA) on the ETDRS chart, intraocular pressure, slit-lamp examination, and SD-OCT. Based on these examinations, one of the investigators (SNB and AGB) decided to switch to aflibercept. Visual acuity and SD-OCT were repeated on a TAE basis after switching to aflibercept.

The following data were collected: time interval between injections, number of injections, BCVA, and (CRT) assessed by SD-OCT.

The primary endpoint was the interval between aflibercept injections after switching and comparing the previous interval between ranibizumab injections.

Secondary endpoints were changes in BCVA and CRT after switching to aflibercept.

Statistical analysis was performed using a Wilcoxon test with GraphPad software (Statview® 1998, SAS Institute Inc.) A *p* value < 0.05 was considered statistically significant.

## 3. Results

### 3.1. Patients Demographic and Baseline Characteristics

Eleven eyes of 11 patients (2 women and 9 men) were included. Patient mean age was 67.1 years (range, 44 to 83 years). Eight out of the 11 patients had a well-perfused form of CRVO, while 3 patients had a mixed CRVO form with ME and peripheral ischemia requiring panretinal photocoagulation. Four eyes (36%) had previously been treated for open-angle glaucoma. Three patients (27%) had previously been diagnosed with high blood pressure, 3 (27%) with cardiac arrhythmia, and one (9%) with type 1 diabetes with mild nonproliferative diabetic retinopathy before CRVO occurrence.

Before switching to aflibercept, 3 patients had previously been switched to dexamethasone implant (Ozurdex) (1–3 injections), but they were rapidly switched back to ranibizumab because of raised intraocular pressure. They were then switched to aflibercept when it became available in France. The data are summarized in Table 1.

### 3.2. Interval between Injections

Before switching, patients received a mean number of 15.3 ± 9.8 ranibizumab injections (range, 6 to 34 injections) during a mean follow-up of 23.4 ± 15.9 months (range, 6 to 57 months). Related to the treatment duration and the TAE regimen, the mean ranibizumab injection interval acquired was 6.1 ± 1.4 weeks (range, 4 to 8 weeks) and remained stable for each patient but could not be more extended unless ME recurrence. Only one patient had a 4-week interval treatment and showed anatomic and functional improvement but with persistence of macular fluid. Thus, he was switched to aflibercept after 6 months and 6 ranibizumab injections. He showed a complete resolution of fluid in the macula with aflibercept and could be progressively extended to a 10-week interval treatment.

**Figure 1:**
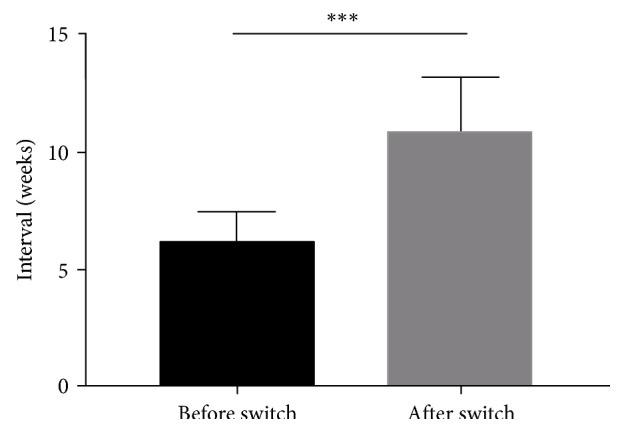
Injection interval before and after the switch to aflibercept. The error bars are interquartile range.

After switching to aflibercept, patients received a mean number of 12.4 ± 4.2 injections (range, 6 to 20 injections) during a mean follow-up of 25.5 ± 5.8 months (range, 16 to 38 months). Due to the switch to aflibercept, the treatment interval was extended from 6.1 ± 1.4 weeks (range, 4 to 8 weeks) to 11 ± 2.3 weeks (range, 8 to 16 weeks) (*p*=0.001) corresponding to a treatment interval gain of 4.9 ± 1.9 weeks (range, 2 to 8 weeks) (Figure 1).

Among patients who were switched to aflibercept, 100% responded to ranibizumab, but 27% (3 patients) only partially responded with persistent fluid on SD-OCT. After switching to aflibercept, 100% of patients responded to aflibercept, and 90% (10 patients) experienced a complete fluid resorption seen on SD-OCT with restoration of a foveal pit. The only patient with persistent macular thickening also had a significant epimacular membrane but no persistent cystoid space left or serous detachment was observed, and the CRT was significantly decreased after the switch to aflibercept.

### 3.3. Functional Outcomes

The initial BCVA before any intravitreal injection was of 41.1 ± 20.9 letters (range, 2 to 65 letters). The mean BCVA before the switch was of 60.5 ± 13 letters (range, 35 to 75 letters), corresponding to a visual gain of +19.4 letters (*p*=0.033). The mean BCVA at the end of follow-up after switching to aflibercept was of 62 ± 16.6 letters (range, 20 to 81 letters) corresponding to a visual gain of +1.5 letters due to the switch (*p*=0.84).

### 3.4. Anatomical Outcomes

**Figure 2:**
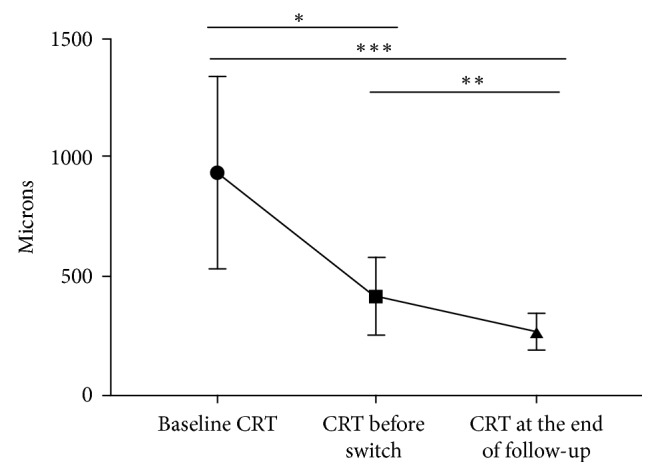
Change in central retinal thickness (CRT) before any injection (baseline), just before the switch to aflibercept, and after the switch at the end of follow-up.

The mean initial CRT before any treatment was 936 ± 402.5 *µ*m. The mean CRT before switching to aflibercept was 411.2 ± 161.8 *µ*m, and it significantly decreased to 264.4 ± 74.3 *µ*m (*p*=0.002) at the end of follow-up (Figure 2).

## 4. Discussion

In this retrospective study, we showed that, in selected cases of ME due to CRVO with frequent recurrences or insufficient anatomical response to ranibizumab with TAE regimen, switching to aflibercept could significantly improve anatomical outcomes (−146.8 *µ*m) and extend the injection interval by 4.9 weeks with a sustained visual acuity gain after a mean follow-up of 25.5 ± 5.8 months (range, 16 to 38 months) after the switch.

Few studies in the literature have focused on visual and functional outcomes after switching from ranibizumab to aflibercept in ME due to CRVO. A retrospective study assessing 6 consecutive eyes with persistent ME despite intravitreal injections of ranibizumab or bevacizumab, conducted in 2014 [13] with a 7-month follow-up, has shown a complete fluid resolution after 1 or 2 aflibercept injections with a modest but sustained visual gain in 3 out of the 6 patients. Another retrospective study [14] assessing 13 eyes with CRVO with insufficient response to ranibizumab or bevacizumab injected every 6 weeks that were switched to aflibercept on a TAE basis has shown that the mean injection interval significantly increased by 0.51 months after 1-year follow-up and that the relapse-free interval also significantly increased by 3.02 weeks, with significantly improved functional and anatomical outcomes. Another retrospective study [10], assessing 17 eyes with ME due to CRVO resistant to bevacizumab or ranibizumab, has shown a significant functional and anatomical improvement after switch to aflibercept. In the later study, a treat-and-extend regimen was used before and after the switch, and the mean interval between intravitreal injections was not extended after the switch. A more recent study [12] assessing 42 eyes with persistent or recurrent ME due to CRVO that received at least 3 ranibizumab and/or bevacizumab intravitreal injections, and then switched to aflibercept, has reported a significant anatomical improvement without significant functional improvement and a significant extension of the interval between injections from 5.6 weeks before the switch to 7.6 weeks after the switch.

Taken together, all these studies and our findings seem to show that switching from ranibizumab/bevacizumab to aflibercept could allow extending the injection interval in a selected population of patients with insufficient anatomical response to ranibizumab or bevacizumab or with frequent recurrences. Despite anatomical improvement, these studies did not show systematic functional improvement. In our series, even CRT improved significantly after the switch, and no functional improvement (BCVA) was observed. This fact is probably caused by morphologic and macula changes in cases with chronic and recurrent ME.

Interestingly, to our knowledge, our study has the longest described follow-up after the switch to aflibercept with a mean of 25.5 ± 5.8 months. The RETAIN study [9] has estimated that, after 48 months of follow-up, 56.2% of patients still required frequent reinjections. Switching to aflibercept could in these selected cases allow reducing the frequency of injections and the treatment burden for both patients and caregivers.

To explain the extension of treatment intervals after switching to aflibercept, we could assume that (i) aflibercept could have a greater affinity for VEGF-A compared to ranibizumab or bevacizumab, (ii) the action of aflibercept not only on VEGF-A but also on PlGF and VEGF-B, could be involved in maintaining its efficacy, (iii) there could be a “switch effect” due to tachyphylaxis developed in response to the first molecule used before the switch, or (iv) the disease could improve by itself over time per se. The latter seems less likely since several studies in the literature have shown an increased treatment interval after switching to aflibercept despite variable follow-up durations before and after the switch. Concerning possible tachyphylaxis to ranibizumab, the treatment frequency did not change before switching to aflibercept in our patients. Ranibizumab treatment interval remained stable but could not be more extended unless ME recurrence. After the switch, the injection interval could be elongated in all our patients, and higher efficacy of the aflibercept treatment could be assumed.

However, to confirm the imputability of aflibercept in this extended relapse-free interval, further controlled studies comparing the efficacy and treatment intervals between ranibizumab and aflibercept are needed. A recent retrospective observational study [16], including 62 naive patients, has shown similar functional and anatomical outcomes after 18 months of follow-up with both treatments with a similar number of injections.

Our study has some limitations, including its retrospective design, a very limited number of eyes, and the absence of the controlled arm. However, our long follow-up after the switch of 25.5 ± 5.8 months (range, 16 to 38 months) confirms a sustained efficacy of aflibercept after the switch at least in terms of anatomical outcomes and treatment interval extension.

In conclusion, in case of insufficient response or frequent recurrences of ME due to CRVO treated with ranibizumab on a TAE basis, switching to aflibercept could allow extending treatment interval, which could reduce the injection burden for these patients. Moreover, after switching to aflibercept despite the absence of significant visual gain, VA stabilization could be expected with a significant anatomical improvement.

## Figures and Tables

**Table 1:** Demographics, baseline, and follow-up characteristics of patients with CRVO.

*Demographics*	
Patients (eyes)	11 (11)
Mean age, years (range)	67.1 (44–83)
Women, *n* (%)	2 (18)
Diabetes, *n* (%)	1 (9)
Diabetic retinopathy, *n* (%)	1 (9)
Preswitch follow-up in months, mean (range)	23.4 (6–57)
Patients previously switched to steroid implant injections, *n* (range of injections)	3 (1–3)
Scatter panretinal photocoagulation, *n* (%)	3 (27%)
Previous ranibizumab injections number, mean ± SD	15.3 ± 9.8
Aflibercept injections number after switch, mean ± SD	12.4 ± 4.2
Postswitch follow-up, in month, mean (range)	25.5 (16–38)

## Data Availability

The data used to support the findings of this study are available from the corresponding author upon request.
